# Cricket Flour for a Sustainable Pasta: Increasing the Nutritional Profile with a Safe Supplement

**DOI:** 10.3390/foods14142404

**Published:** 2025-07-08

**Authors:** Serena Indelicato, Claudia Lino, David Bongiorno, Silvia Orecchio, Fabio D’Agostino, Sergio Indelicato, Aldo Todaro, Lucia Parafati, Giuseppe Avellone

**Affiliations:** 1 Dipartimento di Scienze e Tecnologie Biologiche Chimiche e Farmaceutiche, Università di Palermo, Via Archirafi 32, 90123 Palermo, Italy; serena.indelicato@unipa.it (S.I.); claudia.lino@unipa.it (C.L.); beppe.avellone@unipa.it (G.A.); 2 Dipartimento di Fisica e Chimica, Università di Palermo, Viale delle Scienze, Ed. 17, 90123 Palermo, Italy; silvia.orecchio@unipa.it; 3 Institute of Anthropic Impacts and Sustainability in the Marine Environment (IAS), National Research Council of Italy (IAS-CNR), 91021 Trapani, Italy; fabio.dagostino@cnr.it; 4 Azienda Ospedaliera Ospedali Riuniti Villa Sofia Cervello, Chromatography and Mass Spectrometry Section, Quality Control and Chemical Risk (CQRC), 90146 Palermo, Italy; nondelicato@gmail.com; 5 Department of Agriculture, Food and Environment, University of Catania, Via Santa Sofia, 98, 95123 Catania, Italy; aldo.todaro@unict.it (A.T.); lucia.parafati@unict.it (L.P.)

**Keywords:** cricket flour, protein content, essential amino acids, polycyclic aromatic hydrocarbons, sustainable food, augmented pasta

## Abstract

This study investigates the nutritional and chemical profile of cricket (*Acheta domesticus*) flour, evaluating its potential as a sustainable and highly nutritious food source. Cricket flour, with a protein content of approximately 60%, offers a significantly higher nutritional value compared to many traditional food sources. It is particularly rich in essential amino acids, making it a valuable and sustainable protein alternative. Additionally, the flour is rich in minerals such as potassium, calcium, magnesium, copper, and zinc. The administration of 100 g of cricket flour would exceed the recommended daily intake for adults for most nutrients, making its incorporation into more traditional foods such as bread and pasta at low percentages feasible, easily compensating for any imbalances and increasing their nutritional values. We found that an addition of a mere 10% of cricket flour to produce an experimental pasta fulfilled half of the recommended daily intake values for protein, lipids, and minerals. Chemical analyses of the pure cricket flour revealed only trace amounts of polycyclic aromatic hydrocarbons (PAHs) and linear alkanes, with concentrations well below safety thresholds established for other food categories, indicating that cricket flour is safe for human consumption. The study’s findings confirm that cricket flour is a promising sustainable protein source, and its integration into classic foods could safely contribute to alleviating iron and copper deficiencies as well as malnutrition.

## 1. Introduction

Novel foods, including insect-based products, are regulated under European legislation (Regulation (EC) 258/97) [1] and require prior authorization before commercialization. These products must be properly labeled, indicating species names and potential allergenic risks. In many African, Asian, and Latin American countries, insects are traditionally consumed as food. Their nutritional value and environmental sustainability have attracted growing interest in Western contexts as well. According to the FAO [2], insects offer advantages such as high feed conversion efficiency, rapid growth cycles, and the ability to be reared on organic waste. They emit fewer greenhouse gases than conventional livestock and require significantly less water and space. Nutritionally, insects are rich in high-quality proteins, beneficial fatty acids, fiber, vitamins, and essential minerals such as iron, zinc, and selenium [3,4,5]. These properties make them a promising source of nutrients, particularly in the fight against malnutrition. In addition to their nutritional potential, insect farming is associated with a lower risk of zoonotic disease transmission compared to traditional livestock and is compatible with gluten-free diets. Despite these benefits, insect-derived ingredients such as cricket flour still face cultural resistance in Western societies, often due to food neophobia and unfamiliarity. However, studies have shown that incorporating cricket flour into familiar products like baked goods can improve sensory qualities, such as flavor and aroma, and promote consumer acceptance [6,7,8]. Blending insect-based ingredients with traditional foods may facilitate their gradual integration into the Western diet. Nevertheless, the safe and informed adoption of novel foods requires accurate knowledge of their composition and potential contaminants. This study focuses on cricket (*Acheta domesticus*) flour, commercially available durum wheat flour and pasta as control, and laboratory pasta containing 10% cricket flour. The inclusion percentage was based on literature [9] to reduce adverse effects on sensory acceptance. The aim was to determine key nutritional components, including minerals (Na, K, Ca, Mg), fatty acids, and amino acids. Furthermore, considering that insects may accumulate environmental contaminants either from their habitat or feed [7], the study assessed the presence of other relevant organic substances, such as Total Petroleum Hydrocarbons (TPH), and polycyclic aromatic hydrocarbons (PAHs), along with specific dietary metals (Zn, Cu, Fe).

## 2. Materials and Methods

### 2.1. Reagents and Solvents

All reagents and solvents used were of trace analysis grade and were purchased from Sigma Aldrich (Milan, Italy). Ultrapure water (resistivity: 18 MΩ·cm) was obtained using a Nanopure Diamond purification system (Barnstead International, Waltham, MA, USA).

### 2.2. Samples

Cricket flour, a wheat–cricket flour mixture (containing 10% w/w *Acheta domesticus* flour), and wheat flour (used as a reference) were provided by the Department of Agriculture. Additional wheat pasta (used as control, CTR) and wheat flour samples were purchased from the local market. Pasta samples were prepared using a Kenwood Chef 570 (Kenwood, Havant, UK), equipped with a glass bowl, an enameled K whisk, and completely plastic extruder (Kenwood pasta maker, Kenwood, Havant, UK), mixing 450 g flour (durum wheat semolina, Poiatti, Mazara del Vallo, Italy) and 50 g of cricket flour with 220 mL of distilled water for 10 min. The dough was extruded into a fettuccine shape (20 cm long; hole size: 4 mm × 1.0 mm). Use of stainless-steel parts was avoided to reduce metal contamination.

### 2.3. Physico-Chemical Parameters Determination

Samples of CTR and wheat–cricket pasta were tested to determine moisture content and water activity (Aw). The weight was measured using an analytical balance Ohaus Adventurer (Ohaus Europe, Nänikon, Switzerland). Water activity was determined by an AquaSorp Isotherm Generator (Decagon Devices, Munchen, Germany) at 25 °C. All experiments were conducted in triplicate. Moisture content was measured using an automatic moisture analyzer (Gilbertini, Paderno, Italy) at 110 °C.

### 2.4. Ash and Protein Content Determination

Ash content in cricket flour was determined using a platinum crucible. Approximately 5 g of sample was accurately weighed using an Ohaus Explorer analytical balance (OHAUS Europe GmbH, Nänikon, Switzerland) and placed in a muffle furnace (Nabertherm GmbH, Bremen, Germany). The furnace was programmed to reach 550 °C over four hours, then maintain this temperature for an additional five hours, before allowing the samples to cool to room temperature. The crucible containing the ash residue was transferred to a desiccator containing silica gel and left for one hour to stabilize. The ash content was calculated as the difference in weight between the crucible containing ash and the empty crucible. The incineration process was repeated three additional times, reducing the time to reach 550 °C to two hours and maintaining this temperature for four hours during subsequent cycles, until a constant weight was achieved (defined as a weight variation <5% of the ash content). Crude protein measurements on the cricket flour were performed following themethod by Wang et Al. [10] . The protein amount was finally determined by applying the conversion factor of 6.25 to the corresponding nitrogen content.

### 2.5. Metals Determination

To quantify Na, K, Ca, Mg, Zn, Cu, and Fe, the ash obtained (about 200 mg, exactly weighed) was digested in 2 mL of 69% nitric acid (Fluka Suprapur, Fluka, Buchs, Switzerland). The solution was then diluted to 25 mL with distilled water. When necessary, additional dilutions were performed to ensure the metal concentrations fell within the range of the calibration curves. Elemental analysis was carried out using a PerkinElmer Optima 2100 Series Inductively Coupled Plasma Optical Emission Spectrometer (ICP-OES; PerkinElmer, Springfield, IL, USA). Two types of multi-element standard solutions were used for calibration:

(a) Fluka multi-element standard solution 4 for ICP (17 elements including Zn, Cu, Fe, 10–100 ppm) in HNO_3_ 10%;
(b) Fluka multi-element standard solution III for ICP, specifically for Na, K, Ca, and Mg (200–2000 ppm) in HNO_3_ 5%.

ICP-OES operating parameters were as follows: RF power, 1300 W; sample uptake flow rate, 1.5 mL/min; auxiliary gas flow, 0.2 L/min; nebulizer gas flow, 0.8 L/min; argon gas flow, 15 L/min; viewing mode, axial and radial. The emission signals for each metal were acquired at the wavelengths listed in Table 1.

All calibration standards were prepared from the stock solutions, containing 2% HNO_3_. Calibration curves for the various analytes covered concentrations ranging from 500 to 10,000 µg kg^−1^. In all cases, the correlation coefficients (R^2^) of the regression lines exceeded 0.99. For the major elements analyzed in radial viewing mode, the estimated limits of detection (LOD) ranged from 3 to 10 µg/kg, and the limits of quantification (LOQ) ranged from 10 to 33 µg/kg, in accordance with IUPAC guidelines.

### 2.6. Fat Extraction and Determination

Accurate fatty acid composition analysis requires efficient lipid extraction from the food matrix. Among various extraction techniques [11,12,13,14,15,16] (e.g., Folch, Bligh & Dyer, Soxhlet, percolation, maceration, digestion, steam distillation), the method described by Iverson et al. [15] was used, involving a chloroform/methanol/water mixture. The sample was first finely ground using a porcelain mortar. For extraction, 1 g of sample was mixed with 0.5 g of anhydrous sodium sulfate (Carlo Erba, Milan, Italy) to dry it. Extraction was carried out at room temperature using 1 mL of CHCl_3_ (Fisher Chemical) and 2 mL of CH_3_OH (Carlo Erba), followed by the addition of 1 mL of CHCl_3_. The mixture was vortexed for 30 s for each step, then 1 mL of distilled H_2_O was added to purify the solvent extraction by CH_3_OH and polar compounds. After a second vortexing step, the mixture was filtered through a rapid paper filter ( 110 mm, LLG Labware, Meckenheim, Germany ). The filtrate was transferred to a test tube, and the lower chloroform phase, containing the fat fraction, was recovered. The solvent was evaporated under a nitrogen stream to yield an oily residue, which was weighed using an analytical balance to calculate the fat content as a percentage of sample mass. Fat content was finally used to calculate the percentage of energy from fats in the analyzed samples, using the following equation.

$$E\left(\%\right)=\frac{G_{total}\cdot 9\cdot 100}{CAL}$$

where G_total_ represents the total fat content (g/100 g) determined from the analysis of the novel food, and CAL (kcal) indicates the energy content of the sample, based on its nutritional composition [17].

### 2.7. Gas Chromatographic Analysis of Fatty Acid Methyl Esters (FAMEs)

Fatty acids were methylated directly in the injection vial by adding 0.2 mL of 2 M alcoholic potassium hydroxide (KOH) solution to the hexane extract according to the literature [18]. The mixture was vortexed for 30 s and left to react for at least 30 min prior to analysis. A Thermo Fisher TriPlus autosampler (Waltham, MA, USA) was used for injection. GC-MS analysis was performed using a Thermo Fisher TSQ 8000 instrument (Waltham, MA, USA) coupled with a Thermo Trace 1300 gas chromatograph (Waltham, MA, USA). Separation was achieved using a TR-FAME column (100 m × 0.25 mm i.d., 0.25 µm film thickness; Thermo Fisher), with helium as the carrier gas at a flow rate of 0.8 mL/min. The temperature program was as follows: initial temperature of 50 °C (held for 4 min), ramped at 25 °C/min to 185 °C (held for 4 min), then to 210 °C at 2 °C/min (held for 3 min), and finally to 220 °C at 3 °C/min. A final ramp at 15 °C/min raised the temperature to 310 °C, with a total run time of 36 min. The injection volume was 1 µL with a split ratio of 1:25, and the inlet temperature was set to 300 °C.

Identification of FAMEs was carried out using the Supelco 37 Component FAME Mix Standard (Supelco, Bellefonte, PA, USA). The mass spectrometer, equipped with an Electron Ionization (EI) source, operated at 70 eV in combined Selected Ion Monitoring (SIM) mode—monitoring ions at *m*/*z* 55, 57, 69, 74, and 87—and Full Scan (FS) mode (*m*/*z* 50–400). The transfer line and ion source temperatures were set to 290 °C. FAMEs were identified based on retention times and by comparing spectra with the NIST 2015 Mass Spectral Library. Fatty acid content was expressed as a relative percentage of the total fatty acid content. Peak integration was performed using Xcalibur^TM^ 4.7 software (Thermo Scientific^TM^, Waltham, MA, USA).

Based on literature data [19,20], the health-promoting index (HPI), atherogenicity (IA), and thrombogenicity (IT) indices have been proposed as dietary risk indicators of lipids for cardiovascular diseases. These indexes were determined according to the following equations:

HPI = (MUFAs + PUFAs)/[(C12:0 + 4 × C14:0) + C16:0]

IA = [(4 × C14:0) + C16:0 + C18:0]/[MUFAs + PUFAs-n6 + PUFAs-n3]

IT = (C14:0 + C16:0 + C18:0)/(0.5 MUFAs + 0.5 PUFAs-n6 + 3PUFAs-n3 + PUFAs-n3/PUFAs-n6)

HPI scores have been used to assess the general nutritional value of dietary fat, while the IA represents the relationship between the sum of the main saturated fatty acids (SFA) and that of the main classes of monounsaturated (MUFAs) and polyunsaturated (PUFAs) fatty acids. The IT indicates the tendency to form clots in the blood vessels and considers the relationship between the pro-thrombogenic (saturated) and the anti-thrombogenic fatty acids (MUFAs, PUFAs–n6, and PUFAs–n3).

### 2.8. Amino Acid Content Determination

To analyze the amino acid composition of the cricket flour and wheat–cricket flour mixture, standard amino acid solutions were prepared to develop a qualitative and quantitative GC-MS method. Ethyl chloroformate (ECF) was used as the derivatization reagent [10,21]. Approximately 25 mg of the finely ground sample was weighed and transferred into an autoclavable glass vial. For wheat flour samples, 200 µL of 6 M HCl was added to hydrolyze the protein content. After HCl addition, samples were flushed with nitrogen to eliminate the oxygen dissolved in solution to prevent oxidation and then immediately sealed and incubated in an oven at 110 °C for 24 h. After hydrolysis, samples were dried under a nitrogen stream at room temperature and subjected to derivatization.

For derivatization, 300 µL of water and 300 µL of chloroform were added, resulting in a biphasic system. The upper aqueous phase containing amino acids was separated (50 µL), transferred to a vial with an insert, and the pH adjusted using saturated NaHCO_3_ to ensure neutrality or slight alkalinity. Subsequently,

(i) 50 µL of an ethanol/pyridine (4:1) solution (Sigma Aldrich, Milan) was added and mixed using a glass capillary;
(ii) 10 µL of ECF was added slowly (to control effervescence from HCl release);
(iii) 50 µL of a 1% ECF solution in chloroform was added;
(iv) The mixture was neutralized by adding 50 µL of saturated NaHCO_3_ and stirred until CO_2_ effervescence ceased.

After derivatization, the organic (bottom) layer was recovered, dried using anhydrous sodium sulfate, and transferred to a new vial for GC-MS analysis.

GC-MS analysis was performed using a Thermo Fisher ISQ 1310 mass spectrometer coupled with a Thermo Trace 1300 GC. The column used was a Supelcowax 10 (30 m × 0.25 mm i.d., 0.18 µm film thickness; Merck Life Science S.r.l., Milan, Italy). Helium was used as the carrier gas at a flow rate of 1.2 mL/min. The temperature program was 120 °C (held for 2 min), ramped at 4 °C/min to 240 °C, then ramped at 30 °C/min to 280 °C (held for 5 min). Injection volume was 1 µL (split ratio 1:25), and inlet temperature was 280 °C.

Quantitative analysis was based on calibration curves constructed using an amino acid standard mix 79248 (Sigma Aldrich, Milan, Italy), diluted in Milli-Q water, and derivatized with ECF. Calibration standards were prepared at 0.5, 0.25, 0.125, 0.05, and 0.025 mM for each amino acid. The mass spectrometer operated in full scan (FS) mode, and quantification was based on the extracted ion signals (see Table 2 for monitored *m*/*z* values per amino acid).

### 2.9. Stability of Derivatized Amino Acids

To evaluate the stability of the derivatized amino acid solution from flour samples, gas chromatographic analysis was repeated 24 h after the derivatization process. The chromatogram obtained after 24 h was compared to that recorded immediately after derivatization. This test confirmed that the derivatized amino acids remained stable for at least 24 h and should therefore be analyzed within this time frame.

### 2.10. Determination of Chitin

To assess the presence of chitin in cricket flour, Fourier-transform infrared (FT-IR) spectroscopy was performed following appropriate sample pretreatment. Analyses were conducted using a Perkin Elmer (Springfield, Il, USA) Spectrum Two FT-IR spectrophotometer. Approximately 500 mg of the insoluble residue obtained after lipid extraction, as described by Bligh and Dyer [16], was subjected to further processing.

The residue was first dried in an oven, then treated with 4% (w/v) NaOH at 25 °C overnight. Subsequently, 1 M HCl was added to demineralize carbonate compounds, and the sample was left at room temperature for 1 h. Deproteinization was achieved by treating the sample with 5% (w/v) NaOH at 75 °C for approximately 5 h. After cooling, the sample was neutralized with 1 M HCl, washed with distilled water, centrifuged twice at 4000 rpm, and dried again.

Infrared analysis was then performed by recording spectra in transmittance mode from 4000 to 450 cm^−1^, averaging 20 scans at a resolution of 2 cm^−1^. The KBr pellet was prepared by mixing approximately 1 mg of sample with 100 mg of dry KBr powder.

Characteristic bands indicating the presence of chitin were identified in the recorded spectrum (Figure S1). Specifically, two bands at approximately 1715 and 1630 cm^−1^ were attributed to primary amide groups, confirming the presence of α-chitin. In contrast, β-chitin typically exhibits a single amide band around 1650 cm^−1^ [22,23]. Additionally, bands in the region of 3500–3200 cm^−1^ were assigned to hydroxyl groups, consistent with data reported by Hashem Rasti et al. [24,25]. The spectrum also displayed bands at 2921 and 2851 cm^−1^, indicative of CH_3_ groups [26], and a CH bending vibration around 1385 cm^−1^ [27].

### 2.11. Analysis of Total Petroleum Hydrocarbons (TPH)

Approximately 1.5 g of finely ground and accurately weighed sample was mixed with 0.7 g of anhydrous Na_2_SO_4_. The mixture underwent triple extraction with about 3.5 mL of a chloroform–methanol solution (1:2, v/v). Subsequently, 1 mL of distilled water and 1 mL of chloroform were added. After phase separation, the organic layer was split and pulled into a new glass vial and evaporated under a nitrogen stream.

The residue was then subjected to alkaline digestion using 3 mL of 1 M KOH at 80 °C for 3 h. Following digestion, the mixture was extracted twice with dichloromethane (CH_2_Cl_2_). The organic phase, containing the unsaponifiable fraction, was evaporated to dryness and re-dissolved in 1 mL of hexane.

Prior to GC-MS analysis, 5 µL of an internal standard solution (5-α-androstane at 1 µg/mL) was added to the extract. The analysis was performed using a Thermo Fisher DSQ II mass spectrometer coupled with a Trace 1310 GC system. A TG-SQC column (15 m × 0.25 mm i.d., 0.25 µm film thickness) was used, with helium (99.9995% purity) as the carrier gas at a constant flow rate of 1 mL/min.

The oven temperature program was set as follows: initial temperature of 80 °C (held for 4 min), followed by a ramp of 20 °C/min to 320 °C, which was held for an additional 4 min. A 1 µL injection was performed using a 1:2 split ratio; the injector temperature was maintained at 280 °C. The mass spectrometer operated in electron ionization (EI) mode at 70 eV and used Selected Ion Monitoring (SIM) for quantification, monitoring *m*/*z* 71 for TPH and *m*/*z* 231 for the internal standard. The transfer line and ion source temperatures were both set to 280 °C.

Quantitation was achieved using an internal calibration method. Standard solutions of TPH in hexane were prepared at concentrations ranging from 50 to 400 mg/L by serial dilution from an 8000 ppm stock solution (Mineral Oil Standard, Fluka). The calibration curve showed excellent linearity (R^2^ = 0.99). Limits of detection (LOD) and quantification (LOQ), calculated according to IUPAC guidelines, were 0.1 mg/kg and 0.3 mg/kg, respectively.

### 2.12. Analysis of Polycyclic Aromatic Hydrocarbons (PAHs)

The extraction of PAHs from samples was performed using a protocol similar to that described for linear hydrocarbons. Identification and quantification were conducted using a GC-MS method developed in-house. Analyses were carried out on a Thermo Trace GC 1300 system coupled to a Thermo Scientific TSQ 8000 triple quadrupole mass spectrometer (Waltham, MA, USA).

Chromatographic separation was achieved using a TraceGOLD^TM^ TG-XLBMS column (30 m × 0.18 mm i.d., 0.18 µm film thickness, Thermo, Waltham, MA, USA ). The programmable temperature vaporization (PTV) injector was operated in CT splitless mode with the following settings: split ratio 6.7, split flow 40 mL/min, splitless time 2 min, constant flow 0.80 mL/min, sample volume 2.0 µL, and maximum temperature 340 °C.

The oven temperature program was as follows: initial temperature of 40 °C (held for 2 min), ramped at 10 °C/min to 320 °C, then held for 5 min. The MS transfer line and ion source were set to 280 °C and 300 °C, respectively, with electron ionization (EI) used as the ionization method.

The limits of detection (LOD) and quantification (LOQ) for PAHs were determined to be 0.5 µg/kg and 1.5 µg/kg, respectively. For quantitative analysis, calibration curves were constructed using four standard solutions ranging from 0.5 to 50 µg/kg. These were prepared by diluting a certified reference solution containing 16 PAHs (PAH-Mix 9, 100 µg/mL in cyclohexane; Dr. Ehrenstorfer) with hexane.

Identification of PAHs in both standards and sample extracts was based on retention time matching and confirmed by comparing mass spectra with entries from the NIST spectral library.

To assess the potential carcinogenic risk in our samples, PAHs concentrations were converted into toxic equivalency (TEQ) values using toxic equivalency factors (TEFs) based on benzo[a]pyrene according to Ref. [28]. The TEQ was calculated using the equation:

$$EQ=\sum_{i}{\left[PAH\right]}_{i}\times {TEF}_{i}$$

## 3. Results and Discussion

### 3.1. Physico-Chemical Parameters, Minerals and Metals

Gravimetric analysis revealed that the ash content in cricket flour constituted 4.2% of the dry sample weight, a value consistent with previous findings reported for the edible portion of crickets [29]. It was further determined that the moisture content in both CTR and wheat–cricket pasta (after 48 h drying) amounted, respectively, to 14.04% and 11.07%.

The AW values were 0.83 ± 0.01 for control pasta and 0.64 ± 0.01 for wheat–cricket pasta. The water activity (Aw) values obtained for the flours are consistent with those typically observed in dried pasta and other cereal-based products. These values are considered good, as they fall within the recommended range for dry raw materials (generally Aw < 0.6), ensuring product stability and shelf life. When compared to other flours, the Aw values reported are comparable and do not indicate any unusual moisture-related issues. Such levels are low enough to inhibit the growth of most bacteria, yeasts, and molds, thus limiting microbial development and contributing to microbiological safety. From a techno-functional perspective, these Aw values are favorable, as they help preserve the structural integrity and functionality of the flours during storage. Additionally, maintaining low water activity is crucial for avoiding caking, preserving flowability, and ensuring consistent behavior during processing and formulation. The concentrations of individual minerals, expressed as the average of three replicates, are presented in Table 3.

The nutritional relevance of edible insects as a source of essential minerals has been confirmed in several recent studies [30,31,32]. As shown, the mineral composition of the analyzed samples aligns with previously reported values for edible cricket species [30]. In particular, the iron content (10.8 ± 0.6 mg/100 g) is comparable to the concentration reported in other literature for *Acheta domesticus* (ranging from 4.2 to 11.2 mg/100 g) [29,32]. It is also noteworthy that *Acheta domesticus’* iron content is at least on par or higher than the iron content of other common (*Tenebrio molitor*, *Ruspolia differens*) insect-based novel foods [30], but lower than the iron content of *Melanoplus mexicanus s* (32 mg/100 g) [33]. When compared to wheat flour [34], the iron content in cricket flour is nearly tenfold higher (10.8 mg/100 g vs. 1.3 mg/100 g). Given that iron deficiency (recommended daily intake of iron ranges from 9 to 27 mg) [35] is the most prevalent nutritional disorder worldwide—especially among children and women of reproductive age—the inclusion of edible crickets in the diet may offer a promising strategy for addressing anemia-related conditions [36]. In this respect, *Tenebrio molitor* or *Gryllus bimaculatus* flours appear to be less promising matrices. A similar trend is observed for zinc and copper, which are found in wheat flour at concentrations of 0.84 and 1.6 mg/100 g, respectively. [29]. Zinc and copper levels are also comparable with *Tenebrio molitor* content (zinc 11.2–14.1 mg/100 g, copper 2.1–2.4 mg/100 g) [30], and *Ruspolia differens* (zinc 13 mg/100 g, copper 2.5 mg/100 g) [29].

The high potassium (886 mg/100 g) concentration may be attributed to the crickets’ plant-based diet, as plants tend to accumulate potassium from the soil. The recommended daily intake of potassium is approximately 3200 mg for a 70 kg adult. For comparison, wheat flour contains only 133.0 mg/100 g.

Sodium is present at a concentration of 389 mg/100 g. Given that the recommended daily intake for adults ranges from 500 to 2400 mg, this value is relatively high. In contrast, sodium levels in wheat flour are minimal (20 mg/kg).

Cricket flour also showed a calcium content of 973 mg/kg, substantially higher than that of conventional wheat flour (200 mg/kg). Overall, the cricket flour mineral composition is comparable to other insects’ content, and largely above the mineral content of wheat flour, which, on the other hand, compensates for the high content of sodium in the insect flour (that could be detrimental for blood pressure pathologies). Evaluating these results, in perspective, an optimized mixing of wheat and insect flour could represent a valid diet integration and balancing.

### 3.2. Fatty Acids

In this study, we analyzed a novel food product containing cricket flour, and for comparative purposes, also included conventional food items lacking this ingredient. Due to the limited availability of literature on cricket-enriched foods, the fatty acid profiles of standard commercial products were also assessed.

Total fat content varied across the samples: cricket flour contained 11% total fat, while pasta made of mixed wheat cricket flour showed about 2%. This was notably higher than the 1% total fat measured in pasta made exclusively from durum wheat flour, which aligns with previously reported values [37].

Fat content in insects can vary significantly among species, ranging for the *Orthoptera* order from 4.2% to 48% (Table 4). Even within the same species, variability can arise due to physiological adaptations related to environmental conditions, diets, and particularly fluctuations in temperature [38,39]. Additionally, lipid content may change throughout the insect’s life cycle, with larval stages typically exhibiting higher fat levels [39].

Regarding human fat intake, the EFSA expert panel [40] notes that while specific values for individual fatty acids are not mandated, total fat intake should account for between 20% and 35% of total energy (E%) in adult diets. Intakes below E 35% may limit energy availability and hinder weight maintenance.

Estimated energy contributions from fat and SFA were calculated based on a daily intake of 100 g of food products. Consumption of 100 g of pure experimental cricket flour provided an energy contribution of 23% from fats, whereas pasta containing cricket flour resulted in a caloric intake from lipids of 4.8%. In both cases, the caloric intake was higher than that of traditional pasta, which had a lipid energy contribution of 3.6%. Relative percentages of FA are reported in Table 5 in wheat, pure cricket, and mixed cricket–wheat flour.

**Figure 1:**
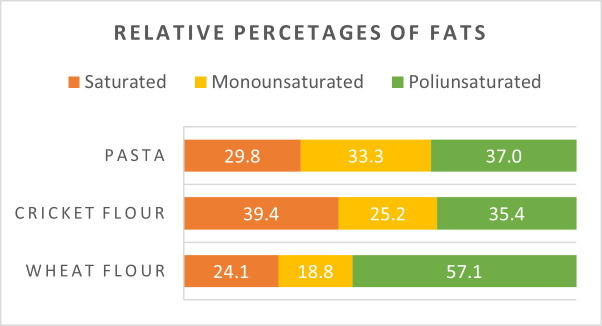
Percentages of fats in different flours investigated.

In our cricket flour, SFA accounted for 42% of total fatty acids, monounsaturated fatty acids (MUFA) for 17%, and polyunsaturated fatty acids (PUFA) for 41%. A comparison of fat compositions across the analyzed food samples is presented in Figure 1.

Numerous epidemiological studies [17] have demonstrated that diets rich in SFAs are associated with increased serum low-density lipoprotein levels and a higher risk of coronary heart disease. Short-chain SFAs (carbon chain length < C10), which are absent in cricket flour, do not raise blood cholesterol levels. Conversely, medium-chain SFAs such as lauric (C12:0), myristic (C14:0), and palmitic acid (C16:0) have been shown to be atherogenic, as first observed by Keys et al. in 1965 [41]. Further studies confirmed that myristic and palmitic acids are also thrombogenic [42], with myristic acid being the most potent cholesterol-raising fatty acid, about four times more than palmitic acid [43].

Myristic acid was detected in the cricket flour samples at a concentration of 2.4%, which is in the same league as stearic acid (C18:0). This acid is less atherogenic due to its rapid conversion to oleic acid in the human body [44]. Palmitic acid (C16:0) was among the most abundant SFAs, representing 34% of total fatty acids in cricket flour, consistent with values reported in the literature (33%) [19].

Although no official maximum intake has been set for SFAs, it is well established that increasing their intake raises the risk of cardiovascular disease. In terms of dietary intake, cricket flour contributes 9.8% of total energy from saturated fats, while pasta with added cricket flour provides 1.9%. Both values fall within the EFSA’s recommended upper limit of 10% of total energy from SFAs.

From a nutritional perspective, MUFAs are associated with beneficial health outcomes, including a reduced risk of cardiovascular disease and inflammation-related disorders [45]. The optimal dietary SFA/UFA (Unsaturated Fatty Acids) ratio is approximately 1:2 [41]. Enriching wheat-based foods with cricket flour could increase the SFA/UFA ratio beyond the optimal range.

Oleic acid, the most nutritionally significant MUFA, was found at 17% in cricket flour, comparable to its levels in traditional wheat pasta (15%) and in pasta enriched with cricket flour (Figure 1). Oleic acid is known to reduce LDL cholesterol and has demonstrated protective effects against several chronic conditions, including cardiovascular diseases, cancer, and neurodegenerative disorders. Like other fatty acids, MUFAs are efficiently absorbed, oxidized for energy, converted into other fatty acids, or incorporated into cellular lipids.

Minor MUFAs such as myristoleic and palmitoleic acids were also identified in cricket flour, at 0.05% and 0.3% of total fatty acids, respectively. These fatty acids are typically absent in wheat flour, as confirmed by literature data [46].

Among polyunsaturated fatty acids, linoleic acid (C18:2, n-6) was predominant in cricket flour, accounting for 39% of total fatty acids. This essential fatty acid cannot be synthesized by humans and must be obtained from the diet. Its deficiency is associated with clinical symptoms such as scaly dermatitis and impaired growth. Linoleic acid is also the precursor of arachidonic acid, which is further metabolized into bioactive eicosanoids.

EFSA recommends that linoleic acid intake should range between 0.6% and 1.2% of total energy [40]. The estimated energy contribution from linoleic acid in cricket flour was 9.1%, whereas in wheat flour it was approximately 1.3% both within or exceeding the recommended values. Increased intake of linoleic acid has been linked to a reduced risk of coronary heart disease [47]. Notably, arachidonic acid and eicosapentaenoic acid were not detected and found at low levels (0.75) in cricket flour.

To assess the healthiness of lipidic fraction of cricket flour some quality indexes (HPI, TI and AI) have been calculated, evidencing that this is still healthy flour, (HPI higher than 1) but presents relatively higher than desired TI and AI indexes, that are mitigated by mixing with wheat flour (Table 5).

Finally, odd-chain fatty acids (e.g., C15:0, C17:0) were identified exclusively in cricket flour, with a mean content of 0.41%. These fatty acids, while less studied, are gaining attention for their potential role in metabolic health.

### 3.3. Amino Acid Profile

Rumpold [29] reported that crickets contain approximately 60–70% protein, without specifying the individual amino acid composition, whereas Payne [46] estimated a lower value of 46%. In the present study, the individual amino acid percentages were determined to evaluate the nutritional profile of cricket flour, with particular emphasis on essential amino acids that cannot be synthesized by the human body.

The total amino acid content in cricket flour, expressed as the mean of three replicate analyses, was found to be 60%, which aligns closely with the results reported by Rumpold and Xiaoming [29,39]. According to the World Health Organization (WHO, 2007) [48], the total daily requirement for amino acids in a 70 kg adult is approximately 58 g, with increased demands under certain physiological conditions. It is worth noting that the protein (60%) content of cricket flour is significantly higher than that of most consumed foods [49], as summarized in Table 6.

**Figure 2:**
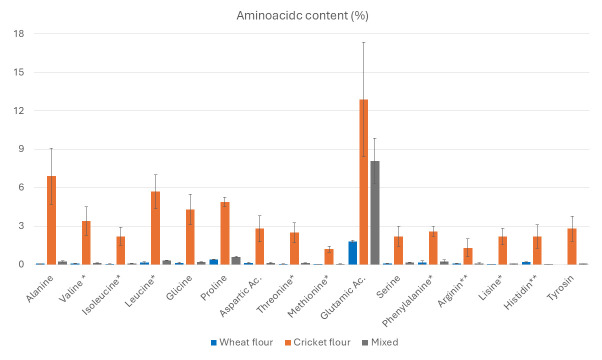
Aminoacidic content (%) in wheat pasta, cricket flour, and mixed flour (wheat–cricket) pasta. (* Essential amino acids, ** Essential aminoacid for children).

As shown in Figure 2, our cricket flour contains substantially higher levels of amino acids compared to wheat flour. Essential amino acids are marked with a single asterisk, and those considered essential for children are indicated with two asterisks.

Assuming a daily intake of 100 g of cricket flour, the intake of each essential amino acid would exceed EFSA’s recommended levels [50]. Since cricket flour can be used in blends with other flours, it may be possible to achieve a balanced amino acid profile in the final product if compositional data are considered during formulation.

The amino acid analysis revealed a marked difference in composition between the cricket flour, the wheat flour, and the mixed sample (10% cricket, 90% wheat) (Table S1). In nearly all measured amino acids, the cricket-based samples displayed significantly higher concentrations compared to the wheat-based ones. For example, the alanine content in the cricket flour exceeded 8 mg/g, while in wheat flour it was only 0.06 mg/g. Similarly, valine concentration was approximately 3.8 mg/g in cricket, but did not exceed 0.11 mg/g in wheat. Comparable trends were observed for isoleucine, leucine, glycine, proline, threonine, methionine, arginine, lysine, and other essential and non-essential amino acids.

In the mixed flour sample, the amino acid content was generally higher than in wheat, though still substantially lower than in pure cricket flour. For instance, threonine concentrations were approximately 0.05 mg/g in wheat, 0.11 mg/g in the mixed sample, and 2.5 mg/g in cricket flour. Similarly, lysine levels increased from ~0.02 mg/g in wheat to ~0.07 mg/g in the mixed sample, while exceeding 2.1 mg/g in the cricket sample.

A one-way ANOVA conducted for each amino acid confirmed that the observed differences among the three groups were statistically significant. In nearly all cases, the *p*-values (Prob > F) were below 0.01, indicating a very low likelihood that the observed differences were due to random variation.

Post-hoc Bonferroni tests were then performed to identify which specific group comparisons were statistically significant. In most cases, cricket flour differed significantly from both wheat and the mixed formulation. Interestingly, wheat flour and the mixed sample were often not significantly different from one another, confirming that the overall amino acid profile in the mixed formulation remained relatively limited at the 10% substitution level.

Some specific amino acids exhibited particularly striking patterns. Phenylalanine levels were nearly 13 times higher in cricket flour compared to wheat. Tyrosine was completely absent from the wheat sample but exceeded 3.7 mg/g in cricket flour. Glutamic acid was also substantially higher in both cricket (9.4 mg/g) and mixed samples (8.1 mg/g) compared to wheat (1.79 mg/g). This, however, could potentially contribute to an umami flavor profile in cricket-enriched products.

Finally, the protein value of *Acheta domesticus* flour should also take into consideration the presence of chitin. Humans can partially digest chitin [51,52] in physiological conditions. Its presence and fermentation have been recently linked to prebiotic function for the human gut, leading to an increased gut biodiversity and, in perspective, suggesting a further nutritional advantage for a healthy diet comprising insect flour.

### 3.4. Hydrocarbon Content and Profiling

**Figure 3:**
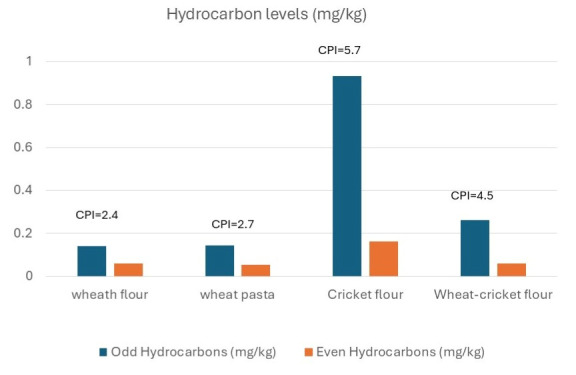
Concentrations of Alkanes with Even and Odd Carbon Numbers.

Cricket flour showed total petroleum hydrocarbon (TPH) concentration, more than twice that observed in the other flour and pasta samples analyzed in this study (Figure 3). Identifying the exact source of these hydrocarbons is difficult without comprehensive information about the farming environment, particularly the quality of the rearing area and insect diet. Insect feeds often include waste materials, and due to their lipophilic nature and high fat content, insects may bioaccumulate non-polar substances such as hydrocarbons.

Currently, no regulatory limits exist for this class of compounds in insect-based foods. However, concentrations reported in some fish species [53] ranging from 1.5 to 18 mg/kg, or pig meat 16–29 mg/kg [54], may serve as reference values. To our knowledge, only one old paper reports information about TPH in *Acheta domesticus* [55]. In our study, the total concentration of linear hydrocarbons (C12–C40) ranged from 0.18 mg/kg in wheat pasta to 1.1 mg/kg in cricket flour, with an intermediate value of 0.32 mg/kg in wheat–cricket blended pasta, values far lower than that reported for fish and meat.

Cricket flour was found to be dominated by the C33 n-alkane, which accounted for 35% of the total linear alkanes. Additional prominent components included C15 (13%), C29 (11%), and C35 (12%).

Based on these data, the Carbon Preference Index (CPI) was calculated as the ratio between odd-numbered and even-numbered n-alkane concentrations. As previously noted [56], combustion sources typically yield a CPI ≈ 1 due to a balanced distribution of hydrocarbon, whereas plant waxes and oils produce odd-carbon-number predominance, resulting in CPI values >1 [56,57,58,59]. On the other hand, meat and meat-related fats are characterized by CPI indexes far lower than 1 [54,60].

All samples showed CPI values above 1, suggesting a predominantly biogenic origin of hydrocarbons. In particular, the high CPI value (5.7) observed in cricket flour suggests a strong biogenic contribution to its hydrocarbon profile, likely from plant-derived compounds. This finding suggests that the hydrocarbon levels found are not due to anthropic activities contamination like combustion [56,57].

### 3.5. Polycyclic Aromatic Hydrocarbons (PAHs)

Table 7 reports the concentrations (in µg/Kg) of individual polycyclic aromatic hydrocarbons (PAHs) and their total content in the analyzed samples. The total PAH concentrations were relatively similar across samples, ranging from 14 µg/Kg in the wheat–cricket flour blend to 27 µg/Kg in wheat pasta.

Phenanthrene was the most abundant compound in all samples, accounting for 66–74% of the total PAHs. Most other PAHs were below the limit of detection (LOD).

Considering the maximum limits set by the European Union [61]—1 µg/kg for the sum of four key PAHs (benzo[a]pyrene, benzo[a]anthracene, benzo[b]fluoranthene, and chrysene) in baby foods, and 35 µg/Kg for smoked bivalve mollusks—it is noteworthy that all analyzed samples, including cricket flour, fall well below these thresholds. As there are currently no specific regulatory limits for insects intended for human consumption, these benchmarks provide a useful point of comparison for further considerations.

In the samples analyzed, TEQ values ranged from 0.02 to 0.04 µg/kg, which are well below the EFSA limits for various food categories (e.g., 1 µg/kg for baby food and 10 µg/kg for smoked seafood) [28,61]. For comparison, benzo[a]pyrene concentrations reported in the literature reach approximately 40 µg/kg in smoked foods and up to 400 µg/kg in grilled products—among the highest levels recorded in foods [62].

Based on data from six European countries, the average adult daily intake (referred to body weight 70 kg) of benzo[a]pyrene is estimated to range between 0.05 and 0.29 µg/day. Consequently, the levels of PAHs observed in our samples suggest no significant health risk and confirm their safety with respect to carcinogenic potential.

In addition to quantification, the relative abundance of specific PAHs can offer insight into their origin. However, source attribution is inherently complex due to the potential contribution of multiple, overlapping, or transient contamination sources, and the possibility of chemical transformation during environmental exposure and metabolic processes.

Nonetheless, specific diagnostic ratios can be informative. A high phenanthrene/anthracene ratio is generally associated with petrogenic (petroleum-derived) sources [63,64], as phenanthrene is more thermodynamically stable. High-temperature processes tend to decrease this ratio due to increased anthracene formation [64,65,66]. Typically, a phenanthrene/anthracene ratio <10 indicates pyrogenic (combustion-related) origins, whereas values >10 suggest petrogenic sources [64].

Similarly, the fluorene/pyrene ratio can help differentiate sources: higher values are linked to combustion processes (coal combustion ≈ 1.4; wood combustion ≈ 1.0) [63], while lower ratios may suggest petroleum contamination. In our study, fluorene/pyrene ratios were close to 1 across all samples, implying a predominant origin from high-temperature combustion.

Phenanthrene/anthracene ratios ranged between 7 and 14, which, although not conclusive, tend to support a mixed origin with significant pyrogenic contribution. In summary, while definitive source identification remains challenging, the observed PAH profiles and diagnostic ratios are consistent with contamination primarily derived from combustion processes rather than petroleum.

### 3.6. Nutritional Aspects

From a nutritional perspective, the recommended daily intake (DI) values established by the Standing Committee on the Scientific Evaluation of Dietary Reference Intakes [67] for a 70 kg adult were used as a reference to evaluate the nutritional potential of cricket flour. Based on the concentrations of key nutrients identified in this study, the estimated amount of cricket flour required to meet DI recommendations varies considerably, as reported in Table 8.

It is evident that, for specific nutrients, such as Ca, Mg, or K, the quantity of cricket flour required to meet DI recommendations would be unrealistically high for routine consumption, whereas for Zn, Cu, and some amino acids, such as Leucine and Threonine the required intake to meet requested DI would by fairly low. However, in practical terms, cricket flour is typically consumed as part of a varied diet. As such, any potential nutritional imbalances can be effectively compensated by other dietary sources.

On the other hand, the intake of approximately 100 g, or even less, of cricket flour is sufficient to meet the DI for total protein and essential amino acids, as well as essential minerals like zinc and copper. This highlights its potential as a valuable dietary supplement, particularly in populations suffering from protein-energy malnutrition or limited access to high-quality protein sources.

It is noteworthy that adding a mere 10% of cricket flour to wheat flour to make pasta reduces by half the dose required to meet the recommended DI for almost all the nutrients considered.

## 4. Conclusions

This study offers a comprehensive evaluation of the nutritional composition and chemical safety of cricket flour, as well as its potential application in fortified food products. The results of the analyses performed on the nutrients demonstrate that cricket flour is a rich source of essential amino acids, surpassing the protein content found in many traditional flours, including meat, cheese, and wheat flour. The amino acid profile of cricket flour is particularly beneficial, as it meets the daily requirements for essential amino acids when consumed in moderate quantities. This makes it a valuable nutritional resource, particularly in contexts where conventional protein sources are limited or unaffordable.

In addition to its high protein content, cricket flour also offers a substantial amount of minerals, including potassium, calcium, magnesium, copper, and zinc. Notably, the addition of just 10% cricket flour (staying within the range of acceptability) to wheat flour in experimental pasta formulations was sufficient to substantially improve the nutritional profile, reducing by half the portion required to meet daily recommendations for several key nutrients. The favorable Aw values, coherent with those typically observed in dried pasta, should also ensure product stability and shelf-life.

Regarding organic pollutant composition occurrence in cricket flour, the study found that polycyclic aromatic hydrocarbons (PAHs) and linear hydrocarbons (as TPH) were present in trace amounts, within safe consumption limits as established for other food categories. Although no specific PAH limits have been set for insects intended for human consumption, the concentrations detected in the samples were far lower than those found in commonly consumed foods, suggesting that cricket flour can be safely included in the diet without posing significant health risks.

Despite all these promising findings, consumer acceptance remains a key barrier to the widespread adoption of insect-based ingredients in Western countries. However, the strategy of incorporating small amounts of cricket flour into familiar foods, such as pasta, and explaining its introduction as a nutritional value added, could lead to a more culturally acceptable transition toward this novel protein source.

## Figures and Tables

**Table 1:** Wavelengths used for the elemental determination by ICP-OES.

Metal	Wavelength 1 (nm)	Wavelength 2 (nm)
Ca	315.887	317.933
Cu	324.752	327.393
Fe	238.204	239.562
K	404.721	766.49
Mg	279.077	285.213
Na	330.237	589.592
Zn	206.204	213.857

**Table 2:** *m*/*z* Values Used for Identification and Quantification of Amino Acids.

Amino Acid	*m*/*z*	Amino Acid	*m*/*z*
Alanine	116, 88, 72, 70	Methionine	61, 129, 175, 101
Valine	114, 116	Glutamic acid	84, 56
Isoleucine	158, 102	Serine	142, 60, 129
Leucine	158, 102	Phenylalanine	102, 91, 176
Glycine	102, 74	Arginine	149, 167
Proline	142, 70	Lysine	156, 128, 84
Aspartic acid	188, 142, 116	Histidine	238, 254, 154
Threonine	129, 101, 74	Tyrosine	264, 192, 107

**Table 3:** Mineral composition of edible cricket flour in milligrams/100 g on dry matter basis (mean ± Standard Error, *p* = 0.05).

	Potassium	Sodium	Calcium	Magnesium	Zinc	Copper	Iron	Literature
Analytes	(mg/100 g)	(mg/100 g)	(mg/100 g)	(mg/100 g)	(mg/100 g)	(mg/100 g)	(mg/100 g)	
*Acheta domesticus* flour (this work)	886 ± 5	389 ± 3	97 ± 1	68 ± 3	22 ± 1	3.4 ± 0.2	10.8 ± 0.6	
*Acheta domesticus* (adult)	1126	435	132–210	80–109	18.6–21.7	0.85–2.01	6.3–11.23	[29]
Tenebrio molitor)	994–1187	159–208	66–142	263–335	11.2–14.1	2.1–2.4	4.5–5	[30]
*Ruspolia differens* (brown adult)	259.7	229	24.5	33.1	12.4	0.5	13	[31]
*Melanoplus mexicanus s*	62	110	120	740	17		32	[31]
*Brachytrupes* sp.			9.2	0.1			0.7	[31]
*Gryllus bimaculatus*	38.6–40.5	166.5	71–74.4		22.4–25	6.8–9.8		[31]

**Table 4:** Lipid Content in Various Insects belonging to the *Orthoptera* order.

Insect	Lipids (g/100 g)	Origin
*Acheta domesticus* flour (this work)	11 ± 2	Italy
*Gryllus assimilis* *	21.8	Brazil
*Acheta domesticus* adult *	22.8	USA
*Acheta domesticus* nymph *	17.7	USA
Melanoplus mexicanus *	4.2	Mexico
*Brachytrupes* sp. *	18.7	Mexico
*Ruspolia differens* *	48.2	Kenya

* From literature data [39].

**Table 5:** Relative percentages of FA in wheat, pure cricket, and mixed cricket–wheat flour (mean ± Standard Error, *p* = 0.05).

	Wheat Flour %	Cricket Flour %	Mixed Flour %
Lauric acid (C12:0)		0.60 ± 0.07	
tetradecanoic acid (C14:0)		2.0 ± 0.2	1.1 ± 0.06
Palmitic acid (C16:0)	24 ± 2	34 ± 3	29 ± 3
Stearic acid (C18:0)		3.1 ± 0.5	
cis-Vaccenic (C18:1-cis)			10.3 ± 0.8
Oleic acid (C18:1-cis)	19 ± 3	25 ± 2	23 ± 2
Linoleic Acid (C18:2-cis 9,12)	57 ± 5	34 ± 3	35 ± 2
Linolenic Acid (C18:2 cis 9,12,15)		1.4 ± 0.1	1.6 ± 0.2
Eicosapentenoic acid (C20:5 cis-5,8,11,14,17)		0.8 ± 0.1	0.10 ± 0.05
Health Promoting Index (HPI)	3.1	1.5	2.1
Trobogenicity Index (TI)=	2.6	2.6	1.8
Atherogeniciy Index (AI)=	0.4	1.2	0.7

**Table 6:** Protein content in selected traditional foods.

Animal-Based Foods	Protein Content (N × 6.25, g/100 g)	Plant-Based Foods	Protein Content (N × 6.25, g/100 g)
Red meat (raw and cooked)	20–33	Vegetables	1–5
Poultry	22–37	Legumes	4–14
Fish	15–25	Fruit	0.3–2
Eggs	11–13	Dried fruit and seeds	8–29
Hard cheeses	27–34	Cooked pasta and rice	2–6
Soft cheeses	12–28	Bread and focaccia	6–13
Dairy products	2–6	Breakfast cereals	5–13

**Table 7:** Concentrations of Polycyclic Aromatic Hydrocarbons (µg/kg, mean ± Standard Error, *p* = 0.05) in pasta and flour samples.

Compound	Wheat Pasta	Wheat Flour	Cricket Flour	Wheat-Cricket Flour
Naphthalene	<LOD	<LOD	<LOD	<LOD
Acenaphthene	<LOD	<LOD	<LOD	<LOD
Acenaphthylene	<LOD	<LOD	<LOD	<LOD
Fluorene	<LOD	<LOD	<LOD	<LOD
Phenanthrene	19 ± 1	12.1 ± 0.7	12.3 ± 0.5	9.3 ± 0.8
Anthracene	1.3 ± 0.4 *	0.9 ± 0.6 *	1.7 ± 0.5	0.7 ± 0.2 *
Fluoranthene	3.5 ± 0.6	1.8 ± 0.3	2.3 ± 0.4	2.1 ± 0.2
Pyrene	3.3 ± 0.8	1.6 ± 0.6	1.8 ± 0.5	1.9 ± 0.2
Benzo[a]anthracene	<LOD	<LOD	<LOD	<LOD
Chrysene	<LOD	<LOD	<LOD	<LOD
Benzo[b]fluoranthene	<LOD	<LOD	<LOD	<LOD
Benzo[k]fluoranthene	<LOD	<LOD	<LOD	<LOD
Benzo[a]pyrene	<LOD	<LOD	<LOD	<LOD
Indeno[1,2,3]pyrene	<LOD	<LOD	<LOD	<LOD
Dibenzo[a]anthracene	<LOD	<LOD	<LOD	<LOD
Benzo[g,h,i]perylene	<LOD	<LOD	<LOD	<LOD
TOTAL	27 ± 2	16 ± 1	18 ± 1	14 ± 1
TEQ	0.04	0.02	0.03	0.02
Fluoranthene/Pyrene	1.1	1.1	1.3	1.1
Phenanthrene/Anthracene	14	14	7	13
Naphthalene	<LOD	<LOD	<LOD	<LOD

(*) Estimated values between LOD and LOQ.

**Table 8:** Nutrient DI recommendations, nutrient content in flours, and intake required to meet the recommended values.

		Cricket Flour		Wheat Flour		Mixed Cricket–Wheat Flour		Mixed vs. Wheat Flour
Analyte	Recommended DI (mg/day)	Content (mg/100 g)	Required DI (g)	Content (mg/100 g)	Required DI (g)	Content (mg/100 g)	Required DI (g)	Ratio
K	3200	886	361	133	2406	194	1646	68.41%
Na	1500	389	386	41	3659	80	1866	51.01%
Ca	1000	97	1028	12	8333	21	4741	56.90%
Mg	350	68	518	68	515	71	495	96.09%
Zn	8.5	22	39	2.8	304	5	179	59.13%
Fe	15	11	139	2.5	600	5	325	54.17%
Cu	1.2	3	35	0.49	245	2	73	29.71%
Lipids	82,700	10,997	752	1000	8270	2000	4135	50.00%
SFA	2750	4583	60	241	1141	596	461	40.44%
Proteins	58,000	61,702	94	3500	1657	10,660	544	32.83%
Valine	2300	3382	68	90	2556	140	1643	64.29%
Isoleucine	1756	2195	80	60	2927	100	1756	60.00%
Leucine	2300	5750	40	180	1278	330	697	54.55%
Threonine	1350	2500	54	60	2250	110	1227	54.55%
Methionine	1300	120	11	10	13,000	50	2600	20.00%

## Data Availability

The original contributions presented in the study are included in the article/Supplementary Material, further inquiries can be directed to the corresponding author.

## Supplementary Materials

The following supporting information can be downloaded at: https://www.mdpi.com/article/10.3390/foods14142404/s1, Figure S1: IR Spectrum of Chitin; Table S1: Aminoacidic content of the investigated flours.
