# Supplementary material for: Cricket Flour for a Sustainable Pasta: Increasing the Nutritional Profile with a Safe Supplement
Source: Foods. 2025 Jul 8;14(14):2404. doi: 10.3390/foods14142404 (PMC12294605; doi:10.3390/foods14142404)
Supplement: Supplementary file 1 [file foods-14-02404-s001.zip › foods-3689380-supplementary.pdf]

Cricket Flour For A Sustainable Pasta: Increasing The Nutri-tional Profile With a Safe Supplement

Serena Indelicato [1], Claudia Lino[1], David Bongiorno [1,*], Silvia Orecchio[2], Fabio D'Agostino[3], Sergio Indelicato[4], Aldo Todaro[5], Lucia Parafati[5] and Giuseppe Avellone[1]

1 Dipartimento di Scienze e Tecnologie Biologiche Chimiche e Farmaceutiche, Università di Palermo, via Archirafi 32, 90123 Palermo, Italy; serena.indelicato@unipa.it (S.I.); claudia.lino@unipa.it (C.L.); david.bongiorno@unipa.it (D.B.); beppe.avellone@unipa.it (G.A.)
2 Dipartimento di Fisica e Chimica, Università di Palermo, viale delle Scienze, Ed. 17, 90123 Palermo, Italy; silvia.orecchio@unipa.it (S.O.)
3 Institute of Anthropic Impacts and Sustainability in the Marine Environment (IAS), National Research Council of Italy (IAS-CNR), Trapani, 91021, Italy; fabio.dagostino@cnr.it (F.D.)
4 Azienda Ospedaliera Ospedali Riuniti Villa Sofia Cervello, Chromatography and Mass Spectrometry Section, Quality Control and Chemical Risk (CQRC), Palermo 90146, Italy; nondelicato@gmail.com (Se.I.)
5 Department of Agriculture, Food and Environment, University of Catania, Via Santa Sofia, 98, Catania, 95123, Italy.; aldo.todaro@unict.it (A.T.); lucia.parafati@unict.it (L.P.)

* Correspondence: david.bongiorno@unipa.it

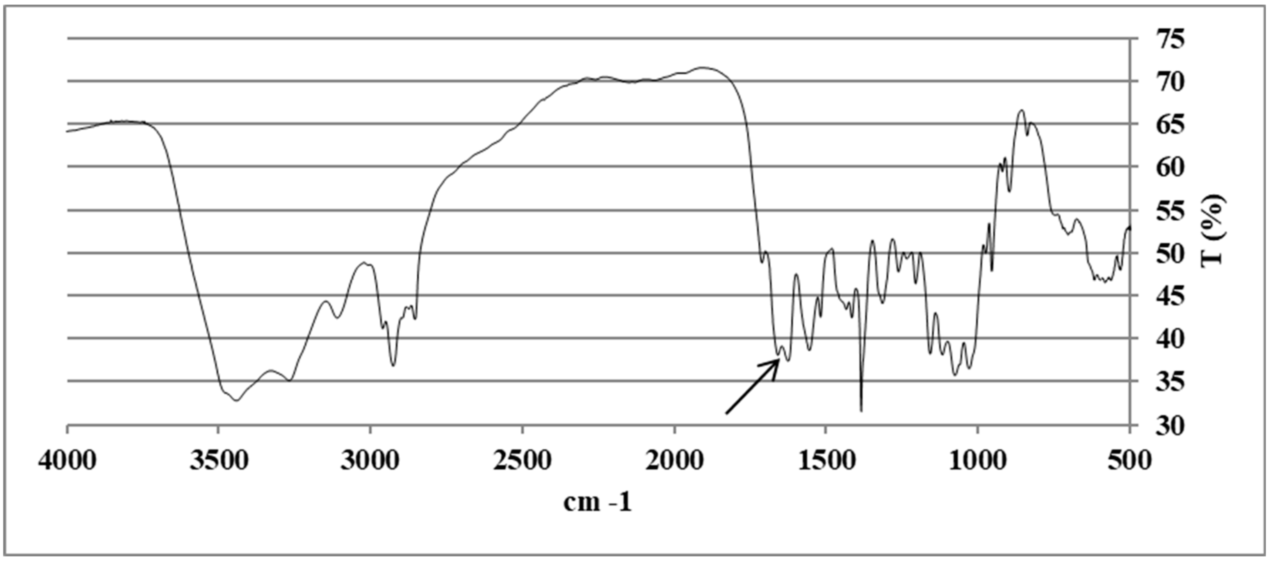

Figure S1: IR Spectrum of Chitin

Table S1: Aminoacidic content of the investigated flours (±standard error, p=0.05)

Aminoacids	Wheat flour			Cricket flour			Mixed flour		
Alanine	0.07	±	0.04	6.9	±	5	0.23	±	0.1
Valine *	0.09	±	0.06	3.4	±	3	0.14	±	0.04
Isoleucine*	0.06	±	0.05	2.2	±	2	0.1	±	0.03
Leucine*	0.18	±	0.1	5.7	±	3	0.33	±	0.06
Glicine	0.12	±	0.2	4.3	±	3	0.21	±	0.04
Proline	0.4	±	0.04	4.9	±	0.9	0.59	±	0.1
Aspartic Ac.	0.13	±	0.1	2.8	±	3	0.13	±	0.10
Threonine*	0.06	±	0.04	2.5	±	2	0.11	±	0.06
Methionine*	0.01	±	0.009	1.2	±	0.6	0.05	±	0.07
Glutamic Ac.	1.8	±	0.2	12.9	±	11	8.09	±	4
Serine	0.1	±	0.03	2.2	±	2	0.17	±	0.04
Phenylalanine*	0.16	±	0.3	2.6	±	0.9	0.26	±	0.3
Arginin**	0.1	±	0.04	1.3	±	2	0.1	±	0.07
Lisine*	0.02	±	0.010	2.2	±	2	0.07	±	0.03
Histidin**	0.2	±	0.08	2.2	±	2	0.01	±	0.005
Tyrosin	0	±	0	2.8	±	2	0.07	±	0.04

*Essential aminoacids, ** essential aminoacids for children.
